# Textural Properties of Chinese Water Chestnut (*Eleocharis dulcis*) during Steam Heating Treatment

**DOI:** 10.3390/foods11091175

**Published:** 2022-04-19

**Authors:** Yu Lu, Siming Zhao, Caihua Jia, Yan Xu, Binjia Zhang, Meng Niu

**Affiliations:** Key Laboratory of Environment Correlative Dietology (Ministry of Education), College of Food Science and Technology, Huazhong Agricultural University, Wuhan 430070, China; aries@webmail.hzau.edu.cn (Y.L.); zsmjx@mail.hzau.ed.cn (S.Z.); xuyan@mail.hzau.edu.cn (Y.X.); zhangbj@mail.hzau.edu.cn (B.Z.); nmjay@mail.hzau.edu.cn (M.N.)

**Keywords:** Chinese water chestnut, thermal treatment, textural properties, correlation

## Abstract

Chinese water chestnut (CWC) has become one of the most popular foods in China. The textural properties of food contribute considerably to consumer preferences. Fresh fruits and vegetables are normally softened after thermal treatment. However, CWC retains most of its crispness and hardness after steaming. To explore the relationship between thermal processes and sensory changes, a method for measuring the texture of CWC is warranted. This study aimed to examine the textural properties of CWC subjected to varying degrees of thermal treatments using instrumental and sensory methods. Instrumental tests included the shear force test and puncture test, while trained panelists assessed the sensory attributes. Two sensory attributes were selected for evaluation: crispness and hardness. The results indicated that with the extension of thermal treatment time, the crispness and hardness of CWC decreased slightly, while cells and starch grains were damaged. Sensory results showed a significant correlation with shear force index (slope of rising curve) (*p* < 0.05) and puncture index (slope of rising curve, slope of descending curve and compression work) (*p* < 0.05). Thereafter, the instrumental tests parameters could be used to establish regression models for predicting crispness and hardness and controlling the quality of CWC products.

## 1. Introduction

Chinese water chestnut (CWC; *Eleocharis dulcis*) is a member of the Cyperaceae, which inhabit swamp waters. Its round edible corm has red-brown peel, with a long brown, triangular appendage. CWC is widely cultivated in southern China and India, and is indigenous to the Hubei, Jiangsu, and Guangdong provinces [1]. In China, the cultivation area of CWC is around 50,000 hm^2^, the output is more than 1.4 million tons, and the output value is more than 14 billion Chinese Yuan [2]. According to traditional Chinese medicine, CWC may be effective in treating hypertension, pharyngitis, and chronic nephritis [3]. Owing to its unique crispy texture and health benefits, the planting area of CWC ranks the third in China, among aquatic vegetables [4].

Texture is the sensory and functional manifestation of the structural, mechanical, and surface properties of food, and is detected through the senses of vision, hearing, touch, and kinesthetics [5]. According to previous research, the textural property of food was an important factor influencing consumer preferences [6]. Crispness, as one of these textural properties, is important to many fruits and vegetables. However, thermal treatments alter the texture of fresh products, making them softer and reducing their crispness [7]. To explore the relationship between thermal processing and the change in crispness, and to find out a more suitable processing method, the texture measurement of CWC is required.

No instrumental determinations could replace human evaluation, which makes sensory determination important for evaluating food quality [8]. However, this is easily affected by the evaluators’ hobbies, emotions, health status, and other factors, and it is therefore difficult to accurately characterize food texture due to limitations of language expression [9,10]. Therefore, some researchers prefer to use instrumental measurements which have greater reproducibility and are faster and less expensive. Non-destructive techniques applied to the evaluation of food texture quality include near-infrared spectroscopy [11,12], machine vision techniques [13], laser-light backscattering imaging [14], nuclear magnetic resonance [15], and ultrasonic techniques [16]. However, non-destructive methods cannot measure food texture directly; thus, their results must be correlated with destructive measurements [17]. At present, a texture analyzer is commonly used to objectively evaluate the texture of fruits and vegetables [18,19,20,21]. To better estimate food texture, several researchers have correlated instrumental analysis data with the sensory evaluation of texture, including bread [22], cheese [23], yogurt [24], and apples [25]. However, it has not been studied to analyze and predict the sensory quality of water chestnut through texture parameters.

In this study, the changes in textural properties and the microstructure of CWC during the steaming process were investigated. The effective instrumental parameter for CWC texture measurement was further determined through analyzing the relationship between instrumental and sensory results. A mathematical model for predicting sensory quality was further established.

## 2. Materials and Methods

### 2.1. Sample Preparation

CWCs were purchased from a local market in Hubei, China. Firstly, CWCs with uniform shapes were selected, cleaned, and peeled. Standard cylinder samples were obtained using a hole punch, nominally 10 mm in diameter. Each cylinder was cut into the length of 10 mm by a mechanically guided razor blade [26]. The pot was then put on the induction cooker (RT2134, Midea group, Foshan, China) and heated with 2000 W power. The heating process was calculated from the boiling. Cylindrical samples were separately steam-heated for 5, 10, 15, 20, 25, and 30 min.

### 2.2. Sensory Evaluation

The hardness and crispness of CWC were evaluated by sensory evaluation. Hardness refers to the force applied by the molar teeth to compress the food and crispness refers to the noise of food during mastication [27]. The panel was composed of five males and five females between the ages of 22 and 24. The panelists preferred CWC. Panelists were trained for 1–2 h each weekend and 35–45 h each year. Each panelist had at least one year of training to recognize hardness and crispness in Table 1. Availability of the panel was judged through the analysis of the number of correct answers over a period of time, while the reliability was evaluated through the repeating test [8].

### 2.3. Instrumental Tests

#### 2.3.1. Shear Force Test

Shear force analysis of CWC was carried out with the TA-XT plus Texture Analyzer (Lotun Science Co., Ltd., Beijing, China). The shear force test can simulate the process of tooth biting samples, and determine the mechanical properties of samples [28,29]. A-LKBF test probe was selected. The test conditions were as follows: test mode: Measure Force in Compression; run: Return to Start; record mode: target. The pre-test speed was set at 1, 3 and 5 mm/s, respectively; the test speed was set at 0.5, 1 and 1.5 mm/s, respectively; the post-test speed was set at 5, 10, and 15 mm/s, respectively; the compression ratio was set at 10, 20, 30%, and the trigger force was set at 30 g. Each working condition was repeated 10 times.

The examination indicators were crispness and hardness. Orthogonal factor level tables were established to analyze the optimal testing conditions by SAS software. The fresh and processed samples were tested under optimized conditions and repeated 15 times in each working condition. The maximum force, compressive work, return work, slope of rising curve, and slope of descending curve were used as evaluation indexes.

#### 2.3.2. Puncture Test

The puncture test can simulate the process of tooth piercing sample, which is generally used to determine the gel strength of samples [30]. In order to produce downward pressure and shear force at the same time, a steel indenter with the certain diameter (smaller than material diameter) was used to pierce the fruit. Therefore, the P6 plate probe (6 mm diameter) was used to test the texture of samples.

The test conditions were as follows: test mode: Measure Force in Compression; run: Return to Start; record mode: target. The pre-test speed was set at 1, 3, and 5 mm/s, respectively; the test speed was set at 1, 3, and 5 mm/s, respectively; the post-test speed was set at 1, 3, and 5 mm/s, respectively; the compression ratio was set at 15%, 20%, 25%, and the trigger force was set at 30 g. Each working condition was repeated 10 times. The analysis of the data was similar to shear force tests.

### 2.4. Observation of CWC Cells

Periodic Acid-Schiff (PAS) stain is mainly used to detect carbohydrates in tissues. Periodic acid oxidized the hydroxyl group to aldehyde group, and then Schiff reagent reacted with aldehyde groups to make them appear purplish red [31]. The stained paraffin sections (Wuhan Google biology Co., Ltd, Wuhan, China) were observed with a fluorescence inverted microscope (Olympus IS70, Tokyo, Japan), and the starch granule morphology as well as cell morphology were recorded.

### 2.5. Data Analysis

The orthogonal experiment results were presented as means ± standard deviation (SD) of at least 5 repeated measurements. The significant differences were evaluated with SPSS Version 24.0 software (SPSS Inc., Chicago, IL, USA). Correlations were estimated with the Pearson test at *p* < 0.05 significance level using SAS Version 9.4 software (SAS Institute, Cary, NC, USA).

## 3. Results and Discussion

### 3.1. Effect of Steaming Time on Sensory Texture of CWC

The hardness of CWC was slightly greater than its crispness, and both showed a decreasing trend with the extension of steaming time (Table 2). Loh cooked CWC with water, steam, and pressure, showing that the fracturability dropped slightly, which was consistent with our study [32]. The highest values for hardness and crispness were found in fresh CWC. Turgor pressure generated by osmosis through the plasma membrane was attributed to this change [33]. However, following steam treatment, the hardness and crispness of CWC remained at the levels of “hard” and “crisp”, respectively. When the cells lost turgor pressure, they could be more easily deformed before being destroyed, and this resulted in a tender texture [34].

### 3.2. Instrumental Tests

#### 3.2.1. Shear Force Test and Puncture Test

The range analysis was used to identify the influence of the test conditions on the standard deviation of the results. The analysis results for shear force test were described in Table 3. According to the value of Ki, the optimal scheme for maximum force was A1B3C3D3; for compression work was A1B1C1D1; for return work was A1B1C1D1; for slope of descending curve was A2B1C1D1; for constant of descending curve was A2B1C1D1.

Summarily, the suitable testing parameters for shear force test were determined as follows through the comparison of the schemes: the pre-test speed was 1 mm/s, the testing speed was 0.5 mm/s, the post-test speed was 5 mm/s, and the compression ratio was 10%.

The analysis results for the puncture test are described in Table 4. According to the value of the Ki, the optimal scheme for maximum force was A2B3C3D3; for compression work it was A2B3C3D3; for return work it was A1B3C3D3; for the slope of the descending curve it was A2B3C3D3; for the constant of the descending curve was A3B3C3D3.

Thereafter, the suitable testing parameters for the puncture test were determined as follows: the pre-test speed was 3 mm/s, the testing speed was 5 mm/s, the post-test speed was 5 mm/s, and the compression ratio was 25%.

#### 3.2.2. Instrumental Determination of CWC at Different Steaming Times

**Figure 1:**
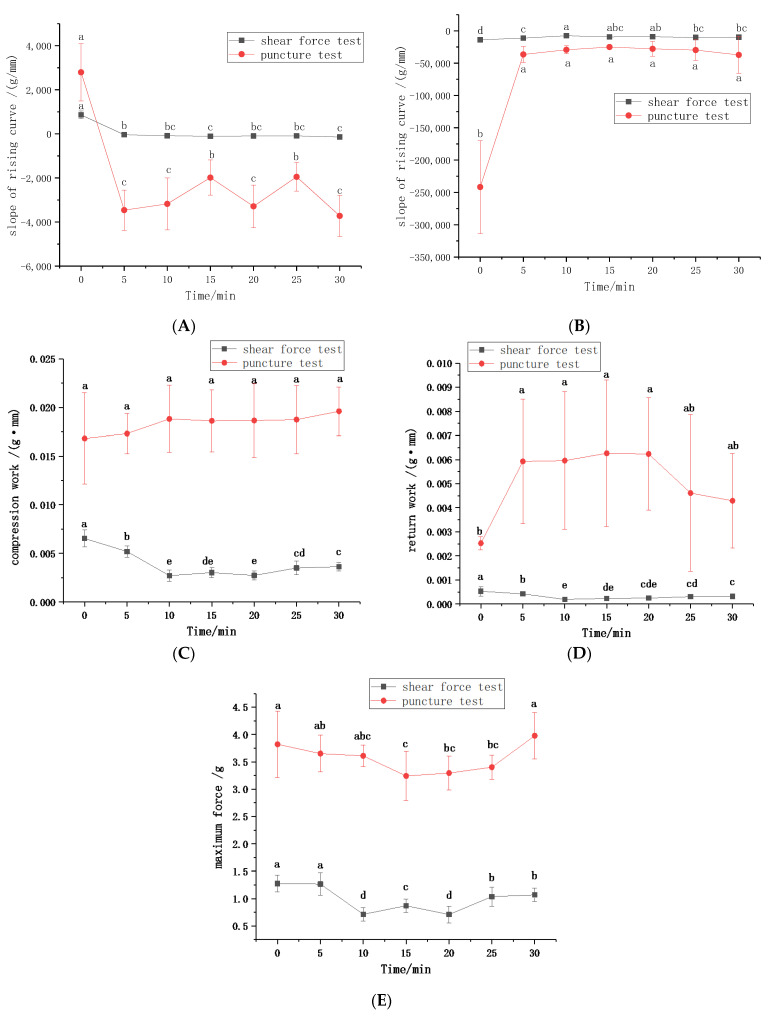
The results of the instrumental test. (**A**) slope of the rising curve; (**B**) slope of the descending curve; (**C**) compression work; (**D**) return work; (**E**) maximum force.

The textural properties of CWC at different steaming times were determined based on the optimization of measurement conditions. Figure 1A shows a plot of the hardness versus steaming time. The hardness of CWC was characterized by the slope of rising curve. Figure 1B illustrates a plot of the crispness versus steaming time. The crispness of CWC was characterized by the slope of descending curve. A smaller slope indicated more brittle CWC. A plot of the compression work versus steaming time is presented in Figure 1C. The area between rising curve and coordinate axis represents compression work. A plot of the return work versus steaming time is described in Figure 1D. The area between the descending curve and coordinate axis meant return work. A plot of the maximum force versus steaming time is reported in Figure 1E, where the maximum force showed strength. Maximum force was a common choice to evaluate hardness and crispness [19,20]; however, it was not very applicable in this experiment. On the contrary, the slope of rising curve and descending curve were more suitable to evaluate hardness and crispness.

### 3.3. Effects of Steaming on the Cell Morphology and the Starch Granule Morphology of CWC

**Figure 2:**
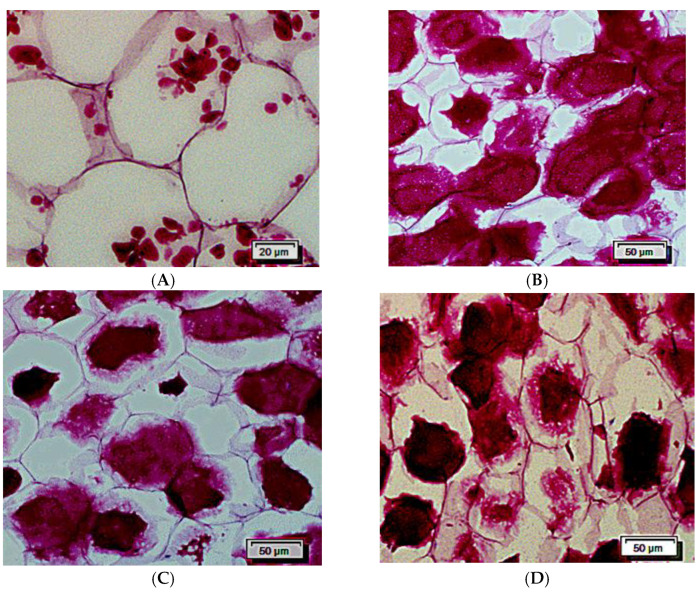
The images of the cell and starch. (**A**) without steaming; (**B**) steaming for 10 min; (**C**) steaming for 20 min; (**D**) steaming for 30 min.

Figure 2A–D show the morphological images of cells and starch granules of CWC under different steaming times. The white parts are the parenchyma cells of CWC, and the red small granules are the starch granules. Figure 2A shows the cells of fresh CWC. The cell diameter of fresh CWC ranged from 60 to 110 μm, with an average diameter of approximately 80 μm, which was nearly eight times larger than starch granules. The cell wall had a clear outline, and a large number of starch granules were unevenly distributed in the cells. In some cells, starch granules were densely distributed, while there were few or no starch granules in others. The thick cell walls may protect CWC from suffering structural destruction during thermal processing [35].

Figure 2B–D show the changes of CWC cells with the extension of steaming time. Following 10 min of steaming treatment, protoplasts of large parenchymal cells in the center of the CWC appeared to shrink. After 20 min of processing, the large parenchymal cells in the center of the CWC were significantly deformed, and their original morphology could not be distinguished. For 30 min of treatment, the outline of the cell wall between the large parenchymal cells in the center of CWC was unclear. Furthermore, the size of melting starch granules was much larger than the original size. The high molecular weight amylopectin could not leach out of the granules, which lead to the inside area of starch granules presenting dark red color. Moreover, the peripheral area of the starch granules with light red may be because of the leaching of amylose and low molecular weight amylopectin [36].

In summary, with prolongation of the steaming treatment, the cell walls gradually shrunk, resulting in the disruption of the cell morphology, and finally cell wall lysis. The sensory texture of hardness and crispness also decreased. This phenomenon was related to the decrease in the swelling force [37], the solubilization of pectic in the middle lamella [38,39,40,41], and the multiple changes of the cell wall matrix components [42].

### 3.4. Correlation Analysis

According to the Pearson’s test, Table 5 presents the significant correlations between the instrumental measures and sensory methods. In the shear force test, the slope of rising curve was positively associated with hardness and crispness, while compression work was positively associated with crispness. In the puncture test, the slope of descending curve was negatively correlated with hardness, and compression work was negatively correlated with crispness. It was also reported that sensory crispness and hardness were strongly and positively correlated with slope of rising curve [25].

On relating the evolution of crispness (C) to change in compression work of puncture test (W_p1_), slope of rising curve of shear force test (K_s1_) and compression work of shear force test (W_s1_), the following regression Equation (1) was obtained:

C = 12.4244 − 318.4434 W_p1_ + 0.0007 K_s1_ + 89.3458 W_s1_, (R^2^ = 0.9167, *p* = 0.0398) (1)

On relating the evolution of hardness (H) to change in slope of rising curve of shear force test (K_s1_), the following regression Equation (2) was obtained:

H = 7.3607 − 0.0035 K_s1_, (R^2^ = 0.8202, *p* = 0.0323) (2)

The results of the multivariate analysis confirmed that the instrumental tests parameters chosen were relevant to the regression models predicting crispness and hardness. The prediction model could calculate crispness and hardness of CWC using the Texture Analyzer.

## 4. Conclusions

The changes of texture, sensory and micromorphology characteristics of CWC under different steam-heating times (0, 5, 10, 15, 20, 25, 30 min) were evaluated for the quality control of CWC products. Steam-heating could reduce the hardness and crispness of fresh CWC, but the extension of steam-heating time had little effect on the change of sensory characteristics of CWC. Meanwhile, it was found that the trend of the slope of rising curve and descending curve were more consistent with the sensory change of CWC. During thermal processing, the morphology of cells and starches was disrupted gradually, and some microstructures may reduce the texture loss caused by thermal process. Additionally, two regression models were developed through the results of Pearson’s test, which provided a more convenient method to evaluate the textural properties of CWC from the industrial and commercial perspectives. Further studies on the changes of some characteristic compounds during thermal processing could be undertaken to comprehensively explore the thermal stability of CWC.

## Figures and Tables

**Table 1:** The descriptions of the texture grades for the CWC.

Texture Grade	Semantic Description	Score
Hardest (Crispest)	The pulp was hard and very juicy	8~10
Harder (Crisper)	The pulp was crisp and juicy	6~8
Hard (Crisp)	The pulp was not crisp like fresh fruits	4~6
Less Hard (Less Crisp)	The pulp chewed somewhat gritty and had a little juice	2~4
Least Hard (Least Crisp)	The pulp chewed like flour and had less juice	0~2

**Table 2:** The effect of steaming time on hardness and crispness of CWC.

Steaming Time	Sensory Score of Hardness	Sensory Score of Crispness
0	9.10 ± 0.74 ^a^	8.30 ± 1.99 ^a^
5	7.10 ± 1.67 ^b^	7.40 ± 1.34 ^ab^
10	7.00 ± 1.58 ^b^	7.00 ± 0.71 ^ab^
15	7.50 ± 1.12 ^b^	6.60 ± 0.55 ^b^
20	6.90 ± 1.02 ^b^	6.40 ± 0.89 ^b^
25	6.20 ± 0.84 ^b^	6.60 ± 1.14 ^b^
30	6.60 ± 0.89 ^b^	6.40 ± 0.55 ^b^

Values followed by different letters are significantly different (*p* < 0.05).

**Table 3:** The range analysis results of shear force test.

		Maximum Force/g	Compression Work/(g·mm)	Return Work/(g·mm)	Slope of Descending Curve/(g/mm)	Constant of Descending Curve/(g/mm)
(A) Pre-test speed/(mm/s)	K1	236.8	652.4	75.2	1416.9	3015.1
	K2	298.4	707.4	99.0	1115.8	2481.4
	K3	253.2	676.1	76.1	1431.1	2994.8
	R	61.5	55.0	23.8	315.3	533.7
(B) Testing speed/(mm/s)	K1	287.7	218.2	54.2	1156.7	1017.6
	K2	305.9	639.4	96.6	1360.3	2832.6
	K3	194.8	1178.2	99.5	1446.9	4641.1
	R	111.1	960.0	45.3	290.2	3623.5
(C) Post-test speed/(mm/s)	K1	287.7	218.2	54.2	1156.7	1017.6
	K2	305.9	639.4	96.6	1360.3	2832.6
	K3	194.8	1178.2	99.5	1446.9	4641.1
	R	111.1	960.0	45.3	290.2	3623.5
(D) Compression ratio/%	K1	287.7	218.2	54.2	1156.7	1017.6
	K2	305.9	639.4	96.6	1360.3	2832.6
	K3	194.8	1178.2	99.5	1446.9	4641.1
	R	111.1	960.0	45.3	290.2	3623.5
optimal experimental conditions		A1B3C3D3	A1B1C1D1	A1B1C1D1	A2B1C1D1	A2B1C1D1

**Table 4:** The range analysis results of the puncture test.

		Maximum Force/g	Compression Work/(g·mm)	Return Work/(g·mm)	Slope of Descending Curve/(g/mm)	Constant of Descending Curve/(g/mm)
(A) Pre-test speed/(mm/s)	K1	530.2	448.6	50.3	41,616.6	48,762.6
	K2	514.4	216.6	299.0	13,216.5	60,765.1
	K3	713.2	387.4	107.2	26,709.7	31,868.0
	R	198.8	231.9	248.7	28,400.1	28,897.2
(B) Testing speed/(mm/s)	K1	987.4	363.0	273.5	50,860.4	106,366.9
	K2	413.8	364.7	94.2	18,865.4	20,568.1
	K3	356.7	324.8	88.7	11,816.9	14,460.7
	R	630.7	39.9	184.8	39,043.5	91,906.2
(C) Post-test speed/(mm/s)	K1	987.4	363.0	273.5	50,860.4	106,366.9
	K2	413.8	364.7	94.2	18,865.4	20,568.1
	K3	356.7	324.8	88.7	11,816.9	14,460.7
	R	630.7	39.9	184.8	39,043.5	91,906.2
(D) Compression ratio/%	K1	987.4	363.0	273.5	50,860.4	106,366.9
	K2	413.8	364.7	94.2	18,865.4	20,568.1
	K3	356.7	324.8	88.7	11,816.9	14,460.7
	R	630.7	39.9	184.8	39,043.5	91,906.2
optimal experimental conditions		A2B3C3D3	A2B3C3D3	A1B3C3D3	A2B3C3D3	A3B3C3D3

**Table 5:** The dependence analysis of the instrumental and sensory assessments.

Index	Slope of Rising Curve/(g/mm)	Slope of Descending Curve/(g/mm)	Compression Work /(g·mm)	Return Work /(g·mm)	Maximum Force/g
Shear force indexes					
Hardness	0.905 ***	−0.724 *	0.717 *	0.646	0.397
Crispness	0.890 ***	−0.782 **	0.889 ***	0.818 **	0.644
Puncture indexes					
Hardness	0.855 **	−0.901 ***	−0.760 **	−0.536	0.242
Crispness	0.783 **	−0.868 **	−0.9038 ***	−0.6022	0.4209

*, **, *** mean the correlation were significant at the 0.1, 0.05, and 0.01, respectively.

## Data Availability

The data presented in this study are available on request from the corresponding author.

## Abbreviations

CWC	Chinese water chestnut

## References

[1] Luo Y., Li X., He J., Su J., Peng L., Wu X., Du R., Zhao Q. (2014). Isolation, characterisation, and antioxidant activities of flavonoids from chufa (*Eleocharis tuberosa*) peels. Food Chem..

[2] Li X., Du Z., Guo X. (2020). Artificial and mechanical harvesting techniques of Chinese water chestnut. J. Chang. Veg..

[3] Zhan G., Pan L., Tu K., Jiao S. (2016). Antitumor, antioxidant, and nitrite scavenging effects of Chinese water chestnut (*Eleocharis dulcis*) peel flavonoids. J. Food Sci..

[4] Man W., Zong Y., Zhao B., Zhu H. (2019). Development status, existing problems and development ideas of aquatic vegetable industry in China. J. Chang. Veg..

[5] Szczesniak A.S. (2002). Texture is a sensory property. Food Qual. Prefer..

[6] Bianchi T., Guerrero L., Gratacós-Cubarsí M. (2016). Textural properties of different melon (*Cucumis melo* L.) fruit types: Sensory and physical-chemical evaluation. Sci. Hortic..

[7] Sila D.N., Smout C., Vu S.T., Van Loey A., Hendrickx M. (2005). Influence of pretreatment conditions on the texture and cell wall components of carrots during thermal processing. J. Food Sci..

[8] Chauvin M.A., Ross C.F., Pitts M., Kupferman E., Swanson B. (2010). Relationship between instrumental and sensory determination of apple and pear texture. J. Food Qual..

[9] Dijksterhuis G., Luyten H., de Wijk R., Mojet J. (2007). A new sensory vocabulary for crisp and crunchy dry model foods. Food Qual. Prefer..

[10] Varela P., Salvador A., Gámbaro A., Fiszman S. (2008). Texture concepts for consumers: A better understanding of crispy—Crunchy sensory perception. Eur. Food Res. Technol..

[11] Hua S.-H., Hsu H.-C., Han P. (2019). P-Wave Visible-Shortwave-Near-Infrared (Vis-SW-NIR) Detection System for the Prediction of Soluble Solids Content and Firmness on Wax Apples. Appl. Spectrosc..

[12] Lan W., Jaillais B., Leca A., Renard C.M., Bureau S. (2020). A new application of NIR spectroscopy to describe and predict purees quality from the non-destructive apple measurements. Food Chem..

[13] Mohamadzadeh Moghadam M., Taghizadeh M., Sadrnia H., Pourreza H.R. (2020). Nondestructive classification of saffron using color and textural analysis. Food Sci. Nutr..

[14] Sanchez P.D.C., Hashim N., Shamsudin R., Nor M.Z.M. (2020). Quality evaluation of sweet potatoes (*Ipomoea batatas L*.) of different varieties using laser light backscattering imaging technique. Sci. Hortic..

[15] Tu S.S., Choi Y.J., McCarthy M.J., McCarthy K.L. (2007). Tomato quality evaluation by peak force and NMR spin-spin relaxation time. Postharvest Biol. Technol..

[16] Contreras M., Benedito J., Garcia-Perez J. (2021). Ultrasonic characterization of salt, moisture and texture modifications in dry-cured ham during post-salting. Meat Sci..

[17] Sirisomboon P., Tanaka M., Kojima T. (2008). Intensive Evaluation of Tomato ‘Momotaro’ Textural Properties. J. Jpn. Soc. Agric. Mach..

[18] Brookfield P.L., Nicoll S., Gunson F.A., Harker F.R., Wohlers M. (2011). Sensory evaluation by small postharvest teams and the relationship with instrumental measurements of apple texture. Postharvest Biol. Technol..

[19] Chiavaro E., Barbanti D., Vittadini E., Massini R. (2006). The effect of different cooking methods on the instrumental quality of potatoes (cv. Agata). J. Food Eng..

[20] Guiné R.P., Barroca M.J. (2012). Effect of drying treatments on texture and color of vegetables (pumpkin and green pepper). Food Bioprod. Processing.

[21] Jaiswal A.K., Gupta S., Abu-Ghannam N. (2012). Kinetic evaluation of colour, texture, polyphenols and antioxidant capacity of Irish York cabbage after blanching treatment. Food Chem..

[22] Santos F.G., Capriles V.D. (2021). Relationships between dough thermomechanical parameters and physical and sensory properties of gluten-free bread texture during storage. LWT-Food Sci. Technol..

[23] Skarlatos L., Marinopoulou A., Petridis A., Raphaelides S.N. (2021). Texture attributes of acid coagulated fresh cheeses as assessed by instrumental and sensory methods. Int. Dairy J..

[24] Skarlatos L., Marinopoulou A., Petridis D., Raphaelides S.N. (2020). Texture assessment of set yoghurt using sensory and instrumental methods. Int. Dairy J..

[25] Bejaei M., Stanich K., Cliff M.A. (2021). Modelling and Classification of Apple Textural Attributes Using Sensory, Instrumental and Compositional Analyses. Foods.

[26] Lázaro A., De Lorenzo C. (2015). Texture analysis in melon landraces through instrumental and sensory methods. Int. J. Food Prop..

[27] Paula A.M., Conti-Silva A.C. (2014). Texture profile and correlation between sensory and instrumental analyses on extruded snacks. J. Food Eng..

[28] Ramana S.V., Wright C.J., Taylor A.J. (1992). Measurement of firmness in carrot tissue during cooking using dynamic, static and sensory tests. J. Sci. Food Agric..

[29] Rahim H.A., Ghazali R., Sahlan S., Maidin M.S. (2013). Prediction of Texture of Raw Poultry Meat by Visible and Near-Infrared Reflectance Spetroscopy. J. Teknol..

[30] Tabilo-Munizaga G., Barbosa-Cánovas G.V. (2005). Rheology for the food industry. J. Food Eng..

[31] Kang H.Q., Chang H.Y., Xu Y.B., Chen F. (2008). Improvement of paraffin section methods and structural observation to endosperm development of rice kernel. Acta Bot Boreal-Occident Sin.

[32] Loh J., Breene W., Davis E. (1982). Between-species differences in fracturability loss: Microscopic and chemical comparison of potato and chinese waterchestnut^1^. J. Texture Stud..

[33] Ranganathan K., Subramanian V., Shanmugam N. (2016). Effect of thermal and nonthermal processing on textural quality of plant tissues. Crit. Rev. Food Sci. Nutr..

[34] Thiel B., Donald A. (2000). Microstructural failure mechanisms in cooked and aged carrots. J. Texture Stud..

[35] Mudahar G.S., Jen J.J. (1991). Texture of raw and canned jicama (*Pachyrrhizus tuberosus*) and Chinese water chestnut (*Eleocharis dulcis*). J. Food Sci..

[36] Fan M., Huang Q., Zhong S., Li X., Xiong S., Xie J., Zhao S. (2019). Gel properties of myofibrillar protein as affected by gelatinization and retrogradation behaviors of modified starches with different crosslinking and acetylation degrees. Food Hydrocoll..

[37] Hai W., Kang T., Wei J., Jikun C. (2007). Texture changes of Chinese water chestnut (*Eleocharis dulcis*) during canning and the relationship between texture and starch properties. Trans. Chin. Soc. Agric. Eng..

[38] Ng A., Waldron K.W. (1997). Effect of cooking and pre-cooking on cell-wall chemistry in relation to firmness of carrot tissues. J. Sci. Food Agric..

[39] Renard C.M. (2005). Effects of conventional boiling on the polyphenols and cell walls of pears. J. Sci. Food Agric..

[40] Sajeev M., Manikantan M., Kingsly A., Moorthy S., Sreekumar J. (2004). Texture analysis of taro (*Colocasia esculenta* L. Schott) cormels during storage and cooking. J. Food Sci..

[41] Waldron K.W., Parker M., Smith A.C. (2003). Plant cell walls and food quality. Compr. Rev. Food Sci. Food Saf..

[42] Van Buren J. (1979). The chemistry of texture in fruits and vegetables. J. Texture Stud..

